# Effects of proline on growth performance, protein synthesis and cold resistance in white shrimp (*Litopenaeus vannamei*)

**DOI:** 10.1016/j.aninu.2025.06.002

**Published:** 2025-07-30

**Authors:** Danyang Zou, Ming Yang, Zhiwen Chen, Pei Lin, Yingtao Li, Xingyuan Liu, Beiping Tan, Chaoxia Ye

**Affiliations:** a Guangzhou Key Laboratory of Subtropical Biodiversity and Biomonitoring, Guangdong Provincial Key Laboratory of Insect Developmental Biology and Applied Technology, Institute of Insect Science and Technology & School of Life Sciences, South China Normal University, Guangzhou 510631, Guangdong, China; b Laboratory of Aquatic Nutrition and Feed, College of Fisheries, Guangdong Ocean University, Zhanjiang 524088, Guangdong, China

**Keywords:** Proline, *Litopenaeus vannamei*, Target of rapamycin signal pathway, Growth performance, Cold stress

## Abstract

This study aimed to explore the effects of proline on growth, protein synthesis, and cold resistance of Pacific white shrimp (*Litopenaeus vannamei*). A total of 720 juvenile shrimp (0.37 ± 0.00 g) were randomly distributed into 18 tanks with 40 shrimp per tank, and the temperature was controlled at 30 ± 1 °C. Shrimp were fed six diets with proline levels of 2.21%, 2.40%, 2.51%, 2.70%, 2.80%, and 3.02%, respectively for 56 d, and then subjected to acute cold stress (20 °C) for 12 h. The results showed that appropriate proline could improve growth performance, feed utilization, free proline, hydroxyproline, glycine, total amino acids, and total non-essential amino acids (*P* < 0.05), as well as mRNA expression (*tor*, *akt,* and *s6k*) and protein level (protein kinase B, Akt) of key genes involved in the target of rapamycin (TOR) signal pathway (*P* < 0.05), suggesting that proline may regulate protein deposition through TOR pathway. Acute cold stress significantly increased hemocyte reactive oxygen species (ROS) level, cell apoptosis and the expression of genes involved in antioxidant (*cat, sod*, *gpx, hsp70*), apoptosis (*caspase-3*, *caspase-9*, *p53, romo1*), autophagy (*lc3*, *atg4*, *becline-1*, and *atg1*), and adenosine 5′-monophosphate (AMP)-activated protein kinase (AMPK)/TOR signaling pathway (raptor and *ampk)*. Under cold stress, dietary proline significantly decreased ROS levels, cell apoptosis and the expression of apoptosis related genes (*caspase-3*, *caspase-9*, and *romo1*), but increased the expression of antioxidant related genes (*gpx* and *hsp70*) and autophagy related genes (*lc3*, *atg4*, *becline-1*, and *atg1*) (*P* < 0.05). In addition, dietary proline significantly up-regulated the mRNA expression of *ampk* and *raptor* and the protein level of phospho-raptor in muscle (*P* < 0.05), while down-regulated the mRNA expression of *s6k* (*P* = 0.024). Based on the quadratic regression analysis of feed conversion ratio, protein efficiency ratio, ROS, apoptosis, and broken line analysis of free proline in muscle, dietary proline content of 2.50% to 2.79% could effectively improve growth, protein synthesis and cold resistance of Pacific white shrimp.

## 1 Introduction

Proline is the only protein-derived secondary amino acid, whose α-amino nitrogen is contained within a pyrrolidine ring. Proline and its metabolite hydroxyproline (Hyp), together with glycine, account for 57% of the total amino acids (AAs) of collagen, and they are crucial for the synthesis of collagen (Li and Wu, 2018). Proline has traditionally been considered a nonessential AA because mammals can synthesize proline from arginine, glutamine and glutamate (Karna et al., 2020). However, the endogenous synthesis rate of proline in pigs, chickens, and fish is insufficient, suggesting that it may be a functional or conditionally essential amino acid (Li and Wu, 2018). Moreover, plant protein sources are low in proline content compared to fish meal (Xie et al., 2015). With the increasing shortage of fish meal resources, plant proteins are more and more widely used in aquatic feed. Thus, it is necessary to evaluate proline nutrition in aquaculture animals. Studies have shown that proline supplement could promote growth of abalone (Vosloo et al., 2013), collagen content in muscle tissue of juvenile turbot (Zhang et al., 2015), muscle quality and antioxidant capability of large yellow croaker (Wei et al., 2020). It has been reported that proline supplementation increased hydroxyproline content in white shrimp (Xie et al., 2015). However, the exact mechanism by which proline exerts beneficial effects on aquatic animals remains largely undetermined.

The target of rapamycin (TOR) is a serine/threonine protein kinase (Battaglioni et al., 2022), mainly present in two different complexes. As the first complex, the target of rapamycin complex 1 (TORC1) is an important growth regulatory molecule that can perceive and interact with various nutritional conditions and environments, including energy levels, growth factors, stress signals, and amino acids (Demetriades et al., 2014; Liu and Sabatini, 2020). Amino acids can enhance the activation of TOR in various cells, thereby activating the activity of TORC1 and increasing protein synthesis in tissues (Bazer et al., 2014; Suryawan et al., 2011; Zhu and Thompson, 2019). The second complex is TORC2, which can influence cell metabolism, survival, proliferation and differentiation by regulating activating protein kinase B (Akt). Its activity is regulated by multiple intracellular factors and it interacts with multiple signaling pathways (Ragupathi et al., 2024). Studies have shown that proline is a multifunctional amino acid (Szabados and Savouré, 2010) that exerts significant effects on protein synthesis, mTOR activation, redox balance, and abiotic stress responses (Wu et al., 2011). For example, proline enhanced the phosphorylation of downstream key protein S6 in the mTOR signaling pathway in rat liver cells, thereby promoting the gap 1/synthesis (G1/S) process in liver cells (Oka et al., 2013). Proline increased the phosphorylation levels of mTOR, p70S6K, and eukaryotic initiation factor 4E-binding proteins (4EBP1) in early weaned piglets, activates the intestinal mTOR pathway, and improved amino acid metabolism and transport (Wang et al., 2021). Additionally, proline could activate the TORC1 signaling pathway in pig trophoblast ectodermal cell line 2 and regulate the intracellular redox environment by enhancing proline transport (Liu et al., 2021). At present, the effect of proline on the TOR signaling pathway in crustaceans is poorly understood.

A large body of evidence suggests that proline metabolism is crucial for cellular stress resistance, but there is little relevant information on crustaceans. Studies have shown that plants, protozoa, and invertebrates accumulated proline in large quantities when they were under stress (Liang et al., 2013; Ren et al., 2018; Surender Reddy et al., 2015; Verbruggen and Hermans, 2008). After acid, alkali, vibrio, and Cd^2+^ stress on white shrimp, it was found that the content of proline in the liver and pancreas increased significantly (Liang et al., 2019). Experimental evidence has indicated that proline supplementation could improve the tolerance of juvenile *Litopenaeus vannamei* to NH_3_ stress (Xie et al., 2015). Water temperature stands as a critical environmental determinant that directly affects the growth, survival, and metabolic processes of white shrimp (Giffard-Mena et al., 2024; Zhou et al., 2011). This recent study found that muscle free proline in white shrimp cultured at long-term low temperature (20 °C) was 1.5 to 1.6 fold higher than that cultured at 28 °C, and metabolomics results showed that most of the upregulated metabolites in hepatopancreas were proline and proline-related dipeptides (Sun et al., 2025), suggesting that proline may be involved in cold tolerance. However, the effect of proline supplementation on cold stress resistance of white shrimp is still unclear.

Pacific white shrimp has broad market prospects and high economic value (Shi et al., 2024), and its production in 2023 was 2.2384 million tons ranking first in crustacean mariculture in China according to China Fishery Statistical Yearbook (2024). The purpose of this study was to investigate the effects of dietary proline on growth performance, free amino acids, TOR signaling pathways and resistance to cold stress in white shrimp. This study may improve the understanding of proline nutrition in crustaceans.

## 2 Materials and methods

### 2.1 Animal ethics statement

This study strictly followed the guidelines of Animal Care and Use Committee of South China Normal University (approval number SCNU-SLS-2023–040).

### 2.2 Experimental design and dietary treatments

Six practical diets were produced by supplementing the basal diet with 0, 0.20%, 0.40%, 0.60%, 0.80%, and 1.00% proline, respectively. The final proline content were 2.21%, 2.40%, 2.51%, 2.70%, 2.80%, and 3.02%, respectively. Details of the proximate analysis for these diets are provided in Table 1. The diets were supplemented with lysine, methionine, L-isoleucine, and phenylalanine to satisfy the requirement for juvenile shrimp. Each feed raw material was crushed and passed through 60-mesh, mixed step by step according to the designed formula table, and distilled water (35% of the feed mixture) was added and mixed evenly. The mixture was processed into pellets with a diameter of 1.2 mm, and subsequently dried to achieve a moisture content of approximately 7%. Then the final diets were enclosed in labeled plastic bags and preserved at −20 °C for subsequent feeding trial.

**Table 1:** Formulation and proximate composition of experimental diets (%, based on dry matter).

Items	Diet 1	Diet 2	Diet 3	Diet 4	Diet 5	Diet 6
**Ingredients**						
Fish meal	11.00	11.00	11.00	11.00	11.00	11.00
Soybean by product	23.00	23.00	23.00	23.00	23.00	23.00
Peanut meal	14.00	14.00	14.00	14.00	14.00	14.00
Rapeseed meal	9.00	9.00	9.00	9.00	9.00	9.00
Chicken meal	4.00	4.00	4.00	4.00	4.00	4.00
Krill meal	5.00	5.00	5.00	5.00	5.00	5.00
Wheat flour	24.10	23.90	23.70	23.50	23.30	23.10
Fish oil	2.50	2.50	2.50	2.50	2.50	2.50
Soybean oil	1.00	1.00	1.00	1.00	1.00	1.00
Phospholipid	1.50	1.50	1.50	1.50	1.50	1.50
Ca(H_2_PO_4_)_2_	2.00	2.00	2.00	2.00	2.00	2.00
Choline chloride (60%)	0.20	0.20	0.20	0.20	0.20	0.20
Vitamin and mineral mixture^1^	1.00	1.00	1.00	1.00	1.00	1.00
Carboxymethyl cellulose	1.00	1.00	1.00	1.00	1.00	1.00
Y_2_O_3_	0.10	0.10	0.10	0.10	0.10	0.10
Cholesterol	0.10	0.10	0.10	0.10	0.10	0.10
Proline	0.00	0.20	0.40	0.60	0.80	1.00
Lysine	0.10	0.10	0.10	0.10	0.10	0.10
Methionine	0.10	0.10	0.10	0.10	0.10	0.10
Phenylalanine	0.20	0.20	0.20	0.20	0.20	0.20
Isoleucine	0.10	0.10	0.10	0.10	0.10	0.10
Total	100.00	100.00	100.00	100.00	100.00	100.00
**Proximate composition**						
Dry matter	93.07	92.95	92.95	92.87	93.01	93.13
Crude protein	43.09	43.53	43.36	43.11	42.96	43.62
Crude lipid	9.55	9.30	9.65	9.57	9.86	9.56
Organic matter	83.97	83.86	83.76	83.94	83.93	84.06
Gross energy, kJ/g	15.18	15.20	15.21	15.23	15.24	15.26

1 Vitamin and mineral mixture ( per kg of mixture): vitamin A, ≥250,000 IU; vitamin D, 35,000 to 200,000 IU; vitamin E, ≥4000 mg; vitamin K_3_, ≥1100 mg; vitamin B_1_, ≥820 mg; vitamin B_2_, ≥800 mg; vitamin B_6_, ≥2500 mg; vitamin B_12_, ≥5 mg; vitamin C, ≥12,000 mg; D-calcium pantothenate, ≥2500 mg; nicotinamide, ≥6000 mg; folic acid, ≥500 mg; D-biotin, ≥10 mg; inositol, ≥3000 mg; magnesium, ≥5000 mg; zinc, 2360 to 15,000 mg; manganese, 630 to 15,000 mg; copper, 170 to 2500 mg; iron, 4500 to 75,000 mg; cobalt, 130 to 200 mg; iodine, 80 to 2000 mg; selenium, 26 to 50 mg; moisture, ≤10% (Guangdong Hyint Biotechnology Group Co., Ltd., Guangzhou, Guangdong, China).

### 2.3 Feeding trial and low temperature challenge

White shrimp were obtained from Guangzhou Jiangmen Shrimp Fry Factory (Guangzhou, Guangdong, China). Ahead of the experiment, shrimp were reared in tanks (300 L) for two weeks to become accustomed to the experimental conditions. Then, 720 healthy shrimp (0.37 ± 0.00 g) were randomly assigned to 18 cylindrical fiberglass tanks (300 L, 3 tanks/diet, 40 shrimp/tank). The shrimp were fed 4 times a day (08:00, 12:00, 17:00, and 22:00) at a fixed point. The shrimp's weight was measured once a week, and the daily feeding amount accounted for 5% to 10% of the shrimp's body weight. The feeding behavior was observed within 2 h after feeding until the shrimp were full. The feeding experiment lasted for 56 d and oxygen was continuously supplied for 24 h. The residue at the bottom of the bucket was collected by siphoning and the uneaten feed was collected by filtration and used to assess the feed intake (FI). Within the experimental timeframe, the ammonia nitrogen (<0.05 mg/L), water temperature (30 ± 1 °C), dissolved oxygen (>6.0 mg/L), pH (7.8 ± 0.2), and salinity ([5 ± 0.5]‰) were adjusted. The entire feeding trial period used alternating natural light. After the completion of the sampling phase within the feeding trial, the remaining shrimp were subjected to acute cold stress (20 °C) for 12 h with three replicates per group, and a portion of shrimp fed diet 1 was kept at 30 °C and used as the control group (2.21% proline).

### 2.4 Sample collection and chemical analysis

At the end of feeding trial, shrimp in each tank were weighed and then sampled for subsequent analysis after 24 h starvation. To assess the whole body composition, five shrimp was selected for analysis in each tank. In addition, six shrimp was randomly taken from each bucket, and their body weight and length were measured to calculate the condition factor (CF). Hepatopancreas and visceral mass were weighed separately to calculate the hepatosomatic index (HSI) and viscerosomatic index (VSI). And hepatopancreas was quickly cut into small pieces and placed in RNA later preservation solution for RNA analysis. Small pieces of muscle were cut and placed in a centrifuge tube for Western blot analysis, and the residual muscle was placed in a sealed bag for free amino acid detection. After the cold stress (20 °C) experiment, three shrimp were randomly selected from each bucket, and their hemolymph was drawn with a 1-mL syringe for flow cytometry analysis. Five shrimp were randomly selected from each bucket, and hepatopancreas and muscle were then removed for subsequent analysis.

The contents of dry matter (method 934.01), crude lipid (method 920.39), crude protein (method 968.06), and ash (method 942.05) in feed and shrimp samples were determined by the AOAC (2005). The content of organic matter (OM) was calculated by the difference between the dry matter and ash content. After drying in an oven (DKN812C, Chongqing Yamato Technology Co., Ltd., Chongqing, China) at 105 °C to constant weight, and the moisture of the samples was measured by calculating the difference. Crude protein was determined by the Dumas's combustion method using a combustion analyzer (D-53639, C. Gerhardt GmbH & Co., KG, Königswinter, North Rhine-Westphalia, Germany). For crude lipid analysis, samples were Soxhlet extracted with petroleum ether. Ash was determined by carbonizing and burning the sample on an electric ceramic furnace, then placing it in a Muffle furnace (FO610C, Yamato Scientific Co., Ltd., Tokyo, Japan), taking it out after 7 h to cool and weigh.

### 2.5 Growth performance and feed utilization

The following variables were calculated:

Weight gain rate (WGR, %) = 100 × (Final body weight – Initial body weight)/Initial body weight;

Specific growth rate (SGR, %/d) = 100 × [Ln (final body weight) - Ln (initial body weight)]/Total experimental days;

Hepatosomatic index (HSI, %) = 100 × (Liver weight/Body weight);

Viscerosomatic index (VSI, %) = 100 × (Visceral weight/Body weight);

Condition factor (CF, g/cm^3^) = 100 × (Body weight/Body length^3^);

Feed intake (FI, g/shrimp) = Total feed intake/Final number of shrimp;

Feed conversion ratio (FCR) = Feed intake/Body weight gain;

Protein efficiency ratio (PER) = Body weight gain/Protein intake;

Protein retention (PR, %) = 100 × (Final body weight × Protein content of final shrimp - Initial body weight × Protein content of initial shrimp)/(Dry weight of feed intake × Feed protein content).

### 2.6 Amino acid composition

The total amino acid composition of diets was determined by the combined method of oxidation and acid hydrolysis, and the results are shown in Table 2. This part was analyzed by Guangzhou Chengyi Aquatic Technology Co., Ltd. (Guangzhou, Guangdong, China).

**Table 2:** Amino acid composition of the experimental diets (%, based on dry matter).

Items	Diet 1	Diet 2	Diet 3	Diet 4	Diet 5	Diet 6
**EAA**						
Arginine	3.00	3.00	3.00	3.00	3.03	3.01
Histidine	1.05	1.04	1.04	1.03	1.06	1.07
Isoleucine	1.71	1.67	1.64	1.63	1.75	1.76
Leucine	3.04	3.03	3.01	3.01	3.08	3.06
Lysine	2.32	2.27	2.24	2.26	2.29	2.36
Methionine	0.90	0.87	0.85	0.86	0.88	0.87
Phenylalanine	2.07	2.06	2.05	2.07	2.10	2.10
Threonine	1.63	1.64	1.62	1.66	1.64	1.64
Valine	2.01	1.99	1.93	1.96	2.12	2.07
ΣEAA	17.73	17.57	17.38	17.48	17.95	17.94
**NEAA**						
Alanine	2.08	2.08	2.08	2.11	2.11	2.10
Aspartic acid	4.22	4.25	4.22	4.26	4.27	4.23
Cysteine	0.60	0.61	0.61	0.60	0.60	0.61
Glutamic acid	7.46	7.47	7.42	7.48	7.49	7.46
Glycine	2.20	2.19	2.18	2.21	2.22	2.21
Proline	2.21	2.40	2.51	2.70	2.80	3.02
Serine	2.09	2.11	2.10	2.14	2.07	2.07
Taurine	0.12	0.12	0.11	0.11	0.11	0.11
Tyrosine	1.39	1.34	1.37	1.34	1.38	1.40
ΣNEAA	22.37	22.57	22.60	22.95	23.05	23.21
ΣTAA	40.10	40.14	39.98	40.43	41.00	41.15

EAA = essential amino acid; NEAA = non-essential amino acid; TAA = total amino acid.

Muscle samples (approximately 100 mg) were accurately weighed for free amino acid determination and transferred into 2.0-mL EP tubes, carefully avoiding residue on the tube walls. In addition, 10% sulfosalicylic acid (CAS#5965–83–3, Tianjin Damao Chemical Reagent Factory, Tianjin, China) was added and thoroughly homogenized with the muscle samples. The mixture was centrifuged in a high-speed refrigerated centrifuge (iCEN-24R, Guangzhou Kepeng Scientific Instrument Co., Ltd., Guangzhou, Guangdong, China) at 13,700 × *g* for 15 min at 4 °C. Following centrifugation, the resulting supernatant was finely filtered through 0.22-μm filter membrane and analyzed using an automated amino acid analyzer (L-8900, Hitachi High-Technologies Co., Tokyo, Japan). Quantitative analysis of amino acids was performed based on peak areas, retention times, and standard curves.

### 2.7 RNA isolation and quantitative real-time PCR (qRT-PCR)

The liver total RNA was extracted from white shrimp that had been rapidly frozen in liquid nitrogen using a kit (74104, Qiagen, Hilden, North Rhine-Westphalia, Germany) in enzyme-free environment. To assess the integrity of RNA, 1.0% agarose gel electrophoresis was conducted, while RNA concentration and purity were determined using a NanoDrop Technologies spectrophotometer (ND-ONE-W, Thermo Fisher Scientific Inc., Wilmington, DE, USA). Subsequently, cDNA was synthesized using a reverse transcription kit (HB210629, Yeasen Biotechnology [Shanghai] Co., Ltd., Shanghai, China).

The cDNA obtained from the above process was used as a template for qRT-PCR. After diluting the primers and samples, the qRT-PCR reaction was performed according to the recommendations of ChamQ SYBR qPCR Master Mix (Q311, Vazyme Biotech Co., Ltd., Nanjing, Jiangsu, China). All procedures were conducted on ice or using low temperature metal ice box. The above procedure was completed in an real-time PCR machine (ABI 7500, Thermo Fisher Scientific Inc., Waltham, MA, USA). After PCR, the amplification and dissociation curves were analyzed to ensure primer specificity and that the PCR reaction met the required standards, thus ensuring the reliability and completeness of the data. The primer information utilized in the study is shown in Table S1. The primers were designed by National Center for Biotechnology Information (NCBI) primer blast. The comparative Ct method (2^−ΔΔCt^) was used to analyse the expression level of relative gene, and *ef1α* was used as reference gene to normalize the results.

### 2.8 Western blotting

The muscle was added to RIPA Lysis Buffer supplemented with PMSF (ST506, Beyotime Biotech Inc., Shanghai, China) and a mixture of protease inhibitors and phosphatase inhibitors. After three rounds of shaking and centrifugating, the supernatant was obtained as the protein solution. The protein concentration of the sample was measured according to the enhanced bicinchoninic acid (BCA) protein assay kit (P0010S, Beyotime Biotech Inc., Shanghai, China), and the sample concentration was uniformly adjusted to 2 μg/μL with ddH_2_O and 5 × sodium dodecyl sulfate polyacrylamide gel electrophoresis (SDS-PAGE) loading buffer. After separating proteins of different molecular weights by SDS-PAGE, the proteins were transferred to polyvinylidene fluoride (PVDF) membrane (IPVH00010, Millipore Sigma, Burlington, MA, USA). Ponceaus S staining solution (P0022, Beyotime Biotech Inc., Shanghai, China) was utilized for the preliminary evaluation of the electrophoresis conditions to determine the successful separation of the target protein and the adequacy of membrane transfer. This membrane was then incubated with antibodies β-actin (Cat#4967), P–S6K (Cat#2708), and p-raptor (Cat#2083), all from Cell Signaling Technology (Danvers, MA, USA); Akt (Cat#10176-2-AP, Proteintech, Rosemont, IL, USA) at 4 °C, followed by washing with Tris-Buffered Saline with Tween 20 (TBST) solution (Biosharp, Hefei Lanjieke International Trade Co., Ltd., Hefei, Anhui, China) to remove the residual primary antibody. Subsequently, it was incubated with an AP-labeled secondary antibody (BA1011, Boster Biological Technology Co., Ltd., Wuhan, Hubei, China) for 1 h at 37 °C. Immune complexes were visualized with an ECL kit (P0018FM, Beyotime Biotech Inc., Shanghai, China). Density analysis of the bands and calculation of grayscale values were performed using ImageJ (National Institutes of Health, Bethesda, ML, USA). In this experiment, each experiment was performed in triplicate to ensure reproducibility, and β-actin was used as a loading control. The reason for this is that expression in shrimp samples is stable and does not overlap with the molecular weight of the protein of interest (Fang et al., 2020; Wei et al., 2023).

### 2.9 Flow cytometry

#### 2.9.1 Detection of hemocyte composition

Firstly, an anticoagulant consisting of 385 mmol/L sodium chloride (CAS#7647–14–5), 115 mmol/L glucose (CAS#50–99–7), and 27 mmol/L trisodium citrate ditydrate (CAS#6132–04–3), all from Tianjin Damao Chemical Reagent Factory (Tianjin, China) was prepared, and the pH reached 7.5. And 100 μL of anticoagulant was aspirated using a 1-mL syringe, followed by the extraction of 100 μL of hemolymph from the pericardial sinus of each shrimp. After 70 mesh cell screening, hemocyte were grouped by a flow cytometer (LSRFortessa X-20, Becton Dickinson and Co., San Jose, CA, USA) (Wang and Chen, 2006).

#### 2.9.2 Determination of intracellular ROS generation

A fluorescent probe DCFH-DA (S0033S, Beyotime Biotech Inc., Shanghai, China) was utilized to assess the generation of ROS by hemocyte. First, DCFH-DA was diluted into 10 μmol/L with anticoagulant, and then the diluted DCFH-DA and hemocyte were configured 1:1 to form the sample working fluid, which was placed in the dark for 30 min. Finally, the intracellular ROS levels were measured using flow cytometry with DCFH-DA fluorescence as an indicator (Chen et al., 2022).

#### 2.9.3 Cell apoptosis assay

Annexin V-FITC apoptosis detection kit (C1062S, Beyotime Biotech Inc., Shanghai, China) was used to detect, and the specific process is as follows: 200 μL hemocyte was taken and placed in the flow tube. 5 μL annexin V, 10 μL propidium iodide (PI) solution, and 185 μL fluorescein isothiocyanate (FITC) binding dilution were added in dark environment. The annexin was mixed and placed in dark environment for 30 min. Finally, the cell apoptosis was detected and analyzed by flow cytometry (Chen et al., 2022).

### 2.10 Statistical analysis

The data were statistically analyzed using the mathematical model for one-way analysis of variance (ANOVA) in Statistical Package for the Social Sciences statistics 26.0 (SPSS Inc., Chicago, IL, USA) as follows:

$$Y_{ij}=\mu +\alpha_{i}+\epsilon_{ij}\text{,}$$

where *Y*_*ij*_ represents the observation from the *j*-th trial within the *i*-th group; *μ* is the overall mean; *α*_*i*_ is the deviation of the mean of the *i*-th group from the grand mean; *ϵ*_*ij*_ is the random error term, which is assumed to be normally distributed with a mean of 0 and variance *σ*^*2*^.

All data were subjected to ANOVA followed by Duncan's multiple range tests. Moreover, a follow-up trend analysis using orthogonal polynomial contrasts was performed to determine whether the significant effects were linear or quadratic. The experimental data were presented as mean and standard error of the mean. *P* < 0.05 was declared as statistically significant and *P* < 0.01 as statistically highly significant.

## 3 Results

### 3.1 Growth performance and feed utilization

The effects of different proline supplemental levels on growth performance and feed utilization of white shrimp are in Table 3. The results showed that significant linear and quadratic effects of proline levels were observed in FCR and PER (*P* < 0.05), and linear effect (*P* < 0.05) was detected in FBW, WGR, SGR, and PR. With the increase of dietary proline from 2.21% to 3.02%, and dietary proline level did not significantly affect CF, HSI, and VSI (*P* > 0.05). The quadratic regression model based on FCR and PER showed that optimal dietary proline requirement for white shrimp at 30 °C was 2.50% to 2.51% (Fig. 5).

**Table 3:** Growth performance and feed utilization of white shrimp fed with different dietary proline levels.

Items	Proline levels, % dry weight						SEM	*P-*value		
	2.21	2.40	2.51	2.70	2.80	3.02		ANOVA	Linear	Quadratic
IBW, g	0.37	0.37	0.37	0.37	0.37	0.37	0.000	0.401	0.414	0.957
FBW, g	15.84^ab^	16.45^a^	15.94^ab^	15.92^ab^	15.42^b^	15.51^b^	0.106	0.041	0.022	0.231
WGR, %	4170.00^ab^	4345.82^a^	4195.60^ab^	4186.71^ab^	4057.10^b^	4080.70^b^	29.409	0.034	0.019	0.233
SGR, %/d	6.70^ab^	6.78^a^	6.71^ab^	6.71^ab^	6.66^b^	6.67^b^	0.012	0.037	0.021	0.236
CF, g/cm^3^	0.84	0.84	0.83	0.83	0.83	0.83	0.004	0.851	0.538	0.517
HSI, %	5.53	5.30	5.53	5.52	5.49	5.55	0.039	0.452	0.453	0.543
VSI, %	6.39	6.23	6.39	6.44	6.38	6.40	0.045	0.874	0.630	0.935
FI, g/shrimp	21.21	21.22	20.79	20.72	21.03	21.17	0.127	0.835	0.800	0.315
FCR	1.38^ab^	1.33^c^	1.35^bc^	1.34^bc^	1.41^a^	1.41^a^	0.009	0.002	0.007	0.003
PER	1.68^abc^	1.73^a^	1.71^ab^	1.73^a^	1.65^bc^	1.62^c^	0.012	0.009	0.013	0.005
PR, %	31.83^ab^	33.37^a^	32.34^ab^	32.20^ab^	30.71^b^	30.51^b^	0.308	0.033	0.012	0.085

IBW = initial body weight; FBW = final body weight; WGR = weight gain; SGR = specific growth rate; CF = condition factor; HSI = hepatosomatic index; VSI = viscerosomatic index; FI = feed intake; FCR = feed conversion ratio; PER = protein efficiency ratio; PR = protein retention; SEM = standard error of the mean.

Values in the same line not sharing a common superscript are significantly different (*P* < 0.05), *n* = 3.

### 3.2 Whole body proximate composition

The effect of dietary proline level on the composition of whole shrimp is shown in Table 4. Dietary proline levels had a significant effect on crude protein in whole shrimp (*P* = 0.023), and the 2.40% proline group showed the highest crude protein, which was similar to the results of growth performance.

**Table 4:** Effects of dietary proline levels on whole body proximate composition of white shrimp (%).

Items	Proline levels, % dry weight						SEM	*P-*value		
	2.21	2.40	2.51	2.70	2.80	3.02		ANOVA	Linear	Quadratic
Moisture	73.38	72.59	73.58	73.63	73.39	73.36	0.135	0.279	0.445	0.844
Crude protein	18.60^ab^	19.04^a^	18.73^ab^	18.70^ab^	18.33^b^	18.33^b^	0.075	0.023	0.013	0.086
Crude lipid	3.20	3.49	3.52	3.39	3.54	3.50	0.059	0.588	0.243	0.364
Crude ash	3.14	3.29	3.07	3.20	3.13	3.19	0.034	0.599	0.989	0.939

SEM = standard error of the mean.

Values in the same line not sharing a common superscript are significantly different (*P* < 0.05), *n* = 3.

### 3.3 Muscle free amino acid

The muscle free amino acid ratios of white shrimp with different proline levels are shown in Table 5. Dietary proline significantly increase the levels of free proline, hydroxyproline, glycine, total nonessential amino acid (NEAA), and total amino acid (TAA) in the muscle of white shrimp (*P* < 0.05). Based on the broken line model of free proline in muscle, the optimal proline requirement in diet of white shrimp was 2.72% (Fig. 5).

**Table 5:** The ratio of free amino acids in muscle of white shrimp fed with different dietary proline levels (mg/g, based on dry matter).

Items	Proline levels, % dry weight						SEM	*P-*value		
	2.21	2.40	2.51	2.70	2.80	3.02		ANOVA	Linear	Quadratic
**EAA**										
Arginine	21.16	21.41	21.62	21.72	21.69	22.14	0.103	0.101	0.006	0.945
Histidine	0.84	0.84	0.86	0.84	0.83	0.83	0.006	0.787	0.484	0.452
Isoleucine	0.37	0.38	0.38	0.36	0.37	0.36	0.003	0.291	0.081	0.558
Leucine	0.75	0.75	0.75	0.75	0.75	0.74	0.006	0.981	0.489	0.727
Lysine	1.15	1.16	1.18	1.19	1.15	1.15	0.012	0.898	0.837	0.413
Methionine	0.21	0.21	0.22	0.21	0.21	0.21	0.002	0.503	0.816	0.778
Phenylalanine	0.34	0.37	0.37	0.36	0.35	0.35	0.005	0.416	0.619	0.074
Threonine	0.30	0.30	0.31	0.31	0.31	0.30	0.004	0.816	0.570	0.354
Valine	0.95	0.92	0.92	0.94	0.92	0.92	0.005	0.431	0.255	0.521
ΣEAA	26.08	26.34	26.61	26.70	26.58	27.00	0.102	0.141	0.011	0.779
**NEAA**										
Alanine	2.56	2.54	2.54	2.52	2.55	2.53	0.021	0.997	0.752	0.930
Cysteine	0.01	0.01	0.01	0.01	0.01	0.01	0.000	0.312	0.373	0.078
Aspartic acid	0.07	0.07	0.07	0.07	0.07	0.07	0.002	0.735	0.575	0.240
Glutamic acid	1.81	2.02	1.95	1.88	1.86	1.79	0.026	0.100	0.219	0.034
Glycine	5.26^b^	5.23^b^	5.34^b^	5.45^ab^	5.43^ab^	5.65^a^	0.042	0.013	0.001	0.267
Hydroxyproline	0.27^d^	0.28^cd^	0.29^c^	0.34^b^	0.34^b^	0.36^a^	0.009	<0.001	<0.001	0.926
Serine	0.53	0.52	0.54	0.53	0.53	0.51	0.005	0.874	0.594	0.448
Proline	30.33^d^	31.77^c^	34.70^b^	36.16^a^	36.48^a^	36.76^a^	0.604	<0.001	<0.001	<0.001
Taurine	1.80	1.82	1.84	1.79	1.80	1.81	0.011	0.909	0.810	0.796
Tyrosine	0.55	0.56	0.58	0.56	0.54	0.57	0.005	0.327	0.805	0.890
ΣNEAA	43.18^d^	44.81^c^	47.86^b^	49.31^a^	49.62^a^	50.06^a^	0.636	<0.001	<0.001	<0.001
TAA	69.26^d^	71.15^c^	74.48^b^	76.01^a^	76.20^a^	77.06^a^	0.703	<0.001	<0.001	<0.001

EAA = essential amino acid; NEAA = nonessential amino acid; TAA = total amino acid; SEM = standard error of the mean.

Values in the same line not sharing a common superscript are significantly different (*P* < 0.05), *n* = 3.

### 3.4 Expression of key genes and proteins in the TOR signaling pathway

**Fig. 1:**
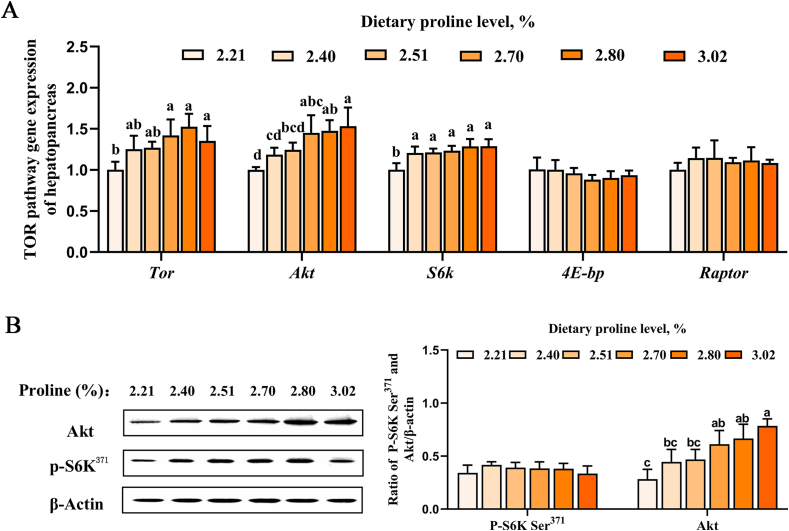
Effects of dietary proline level on (A) **e**xpression of key genes and (B) proteins in the TOR signaling pathway in white shrimp. Akt = protein kinase B; P–S6K Ser^371^ = phospho-p70 S6 kinase (Ser371). Different lowercase letters above columns represent significant differences among treatments at *P* < 0.05.

The expression of genes and proteins related to TOR signaling pathway in white shrimp is shown in Fig. 1. The mRNA expression of *tor*, *akt*, and *s6k* increased significantly with proline levels (*P* < 0.05). As the dietary proline content rises, the protein expression of Akt increased significantly, and it reached the highest when dietary proline content was 3.02% (*P* < 0.05).

### 3.5 Effects of proline on hemocyte composition, ROS, and apoptosis under cold stress

**Fig. 2:**
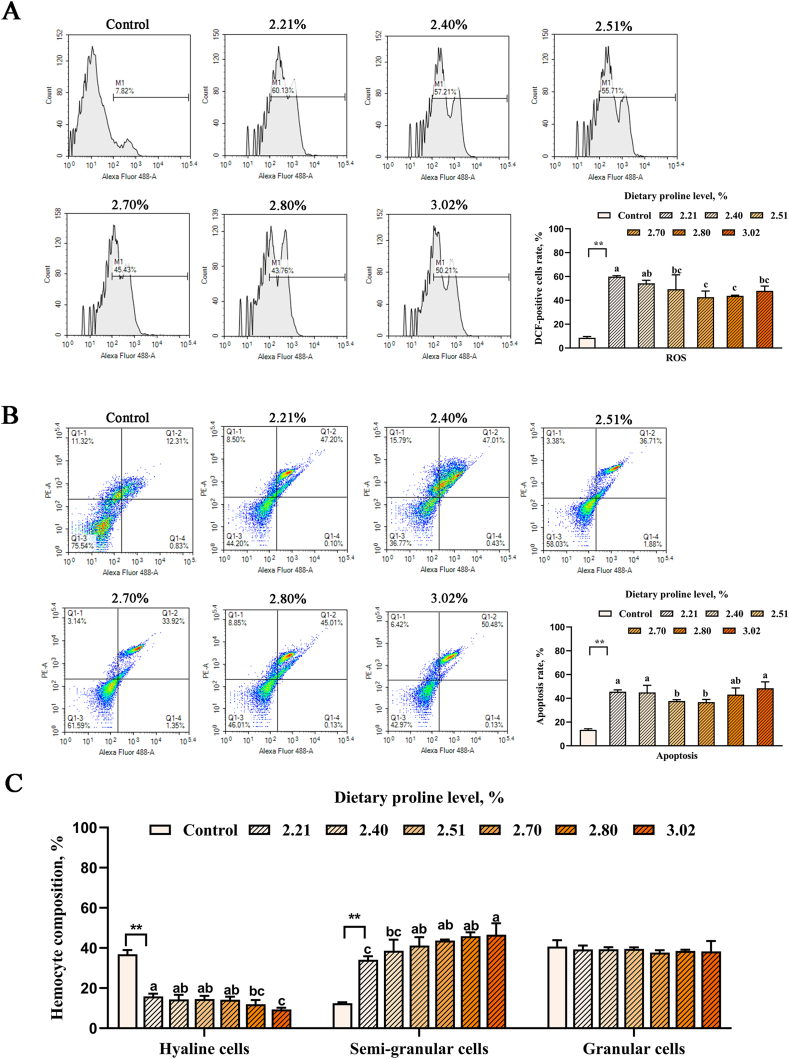
Effects of dietary proline on (A) ROS, (B) apoptosis and (C) hemocyte composition of hemocytes in white shrimp under 20 °C cold stress. Dietary 2.21% proline was used as the control group under 30 °C. Different lowercase letters above columns represent significant differences among treatments at *P* < 0.05. The asterisks (∗∗) indicate that there is a significant difference between the control group and the cold stress group (*P* < 0.01). PE-A = phycoerythrin-area; ROS = reactive oxygen species.

As shown in Fig. 2, after 12 h of acute cold stress, with the increase of proline content, the proportion of hyaline cells in the hemolymph of white shrimp decreased significantly (*P* < 0.05), while the proportion of semi-granular cells increased significantly (*P* < 0.05). Meanwhile, both ROS levels and apoptosis rates decreased and then increased with increasing proline (*P* < 0.05). With the increase of proline content, hyaline cells, ROS, and apoptosis decreased significantly (*P* < 0.05), while semi-granular cells increased significantly (*P* < 0.05). The quadratic regression model based on ROS and apoptosis showed that optimal dietary proline requirement of white shrimp was 2.60% to 2.79% (Fig. 5).

### 3.6 Expression of antioxidant, apoptosis, and autophagy genes under cold stress

**Fig. 3:**
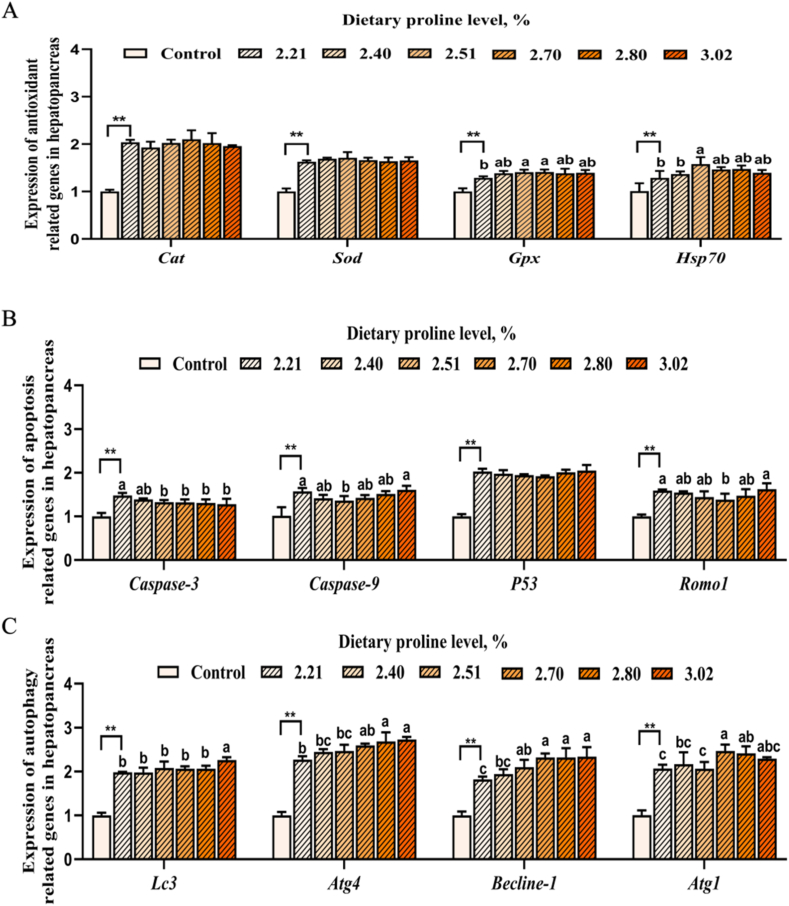
Effects of dietary proline on (A) antioxidant, (B) apoptosis and (C) autophagy related genes in hepatopancreas of white shrimp under 20 °C cold stress. Dietary 2.21% proline was used as the control group under 30 °C. Different lowercase letters above columns represent significant differences among treatments at *P* < 0.05. The asterisks (∗∗) indicate that there is a significant difference between the control group and the cold stress group (*P* < 0.01).

As exhibited in Fig. 3, cold stress significantly increased the mRNA expression of genes involved in antioxidant (*cat, sod*, *gpx,* and *hsp70*), apoptosis (*caspase-3*, *caspase-9, p53*, and *romo1*) and autophagy (*lc3*, *atg4*, *becline-1*, and *atg1*) in hepatopancreas of white shrimp (*P* < 0.05). The mRNA expression of *gpx* and *hsp70* was the highest when dietary proline was 2.51%. The mRNA expression of *caspase-3*, *caspase-9*, and *romo1* reached the minimum value when dietary proline was 2.51% and 2.70% (*P* < 0.05). The mRNA abundance of *atg4*, *becline-1*, and *atg1* increased with increasing dietary proline level up to 2.70% (*P* < 0.05).

### 3.7 Gene and protein expression of TOR signaling pathway under cold stress

**Fig. 4:**
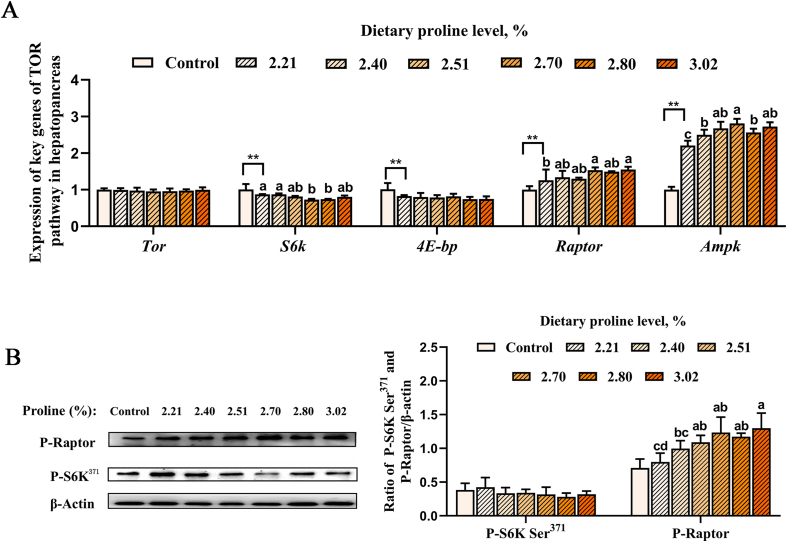
Effects of dietary proline on (A) AMPK/TOR signaling pathway gene and (B) protein expression in white shrimp under 20 °C cold stress. Dietary 2.21% proline was used as the control group under 30 °C. P–S6K Ser^371^ = phospho-p70 S6 kinase (Ser371); P-Raptor = phosphor-raptor. Different lowercase letters above columns represent significant differences among treatments at *P* < 0.05. The asterisks (∗∗) indicates that there is a significant difference between the control group and the cold stress group (*P* ≤< 0.01).

As shown in Fig. 4, after 12 h of cold stress, the mRNA abundance of *s6k* and *4e-bp* decreased significantly, while the gene expression of raptor and *ampk* increased significantly (*P* < 0.05). The mRNA abundance of *s6k* gradually decreased while the genes expression of raptor and *ampk* significantly increased when the dietary proline level increased from 2.21% to 2.70% (*P* < 0.05). As shown in Fig. 4, the protein content of p-raptor in muscle significantly increased with the increase of dietary proline level up to 2.70% (*P* < 0.05).

**Fig. 5:**
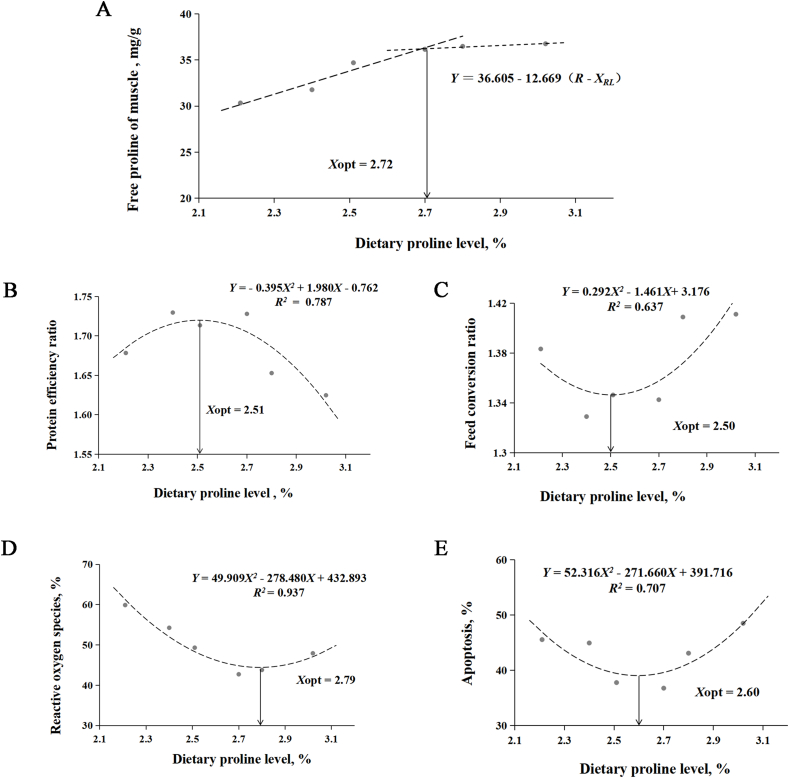
Quadratic regression analysis and broken-line analysis of white shrimp fed different proline levels. (A) The broken-line model for the relationship between free proline in muscle and dietary proline levels in shrimp. (B) The quadratic regression analysis model for the relationship between protein efficiency ratio and dietary proline levels in shrimp. (C) The quadratic regression analysis model for the relationship between feed conversion ratio and dietary proline levels in shrimp. (D) The quadratic regression analysis model for the relationship between reactive oxygen species and dietary proline levels in shrimp. (E) The quadratic regression analysis model for the relationship between apoptosis and dietary proline levels in shrimp. Fig. A-C show the prediction of proline requirement for the 30 °C feeding trial, while Fig. C and D show the 20 °C cold stress, respectively. Xopt = optimal X value.

## 4 Discussion

Proline is considered a multifunctional amino acid that is important for improving animal growth, reproduction, development and promoting protein synthesis (Alvarez et al., 2022; Szabados and Savouré, 2010; Verslues and Sharma, 2010). However, it is still unknown whether proline can be adequately synthesized in aquatic animals to meet the optimal needs for health and growth (Li et al., 2009). In this study, adequate addition of proline to a diet containing a low proportion of fish meal (11%) could significantly improve the performance of white shrimp. Research has demonstrated that supplementation with optimal levels of proline may enhance growth in abalone (Vosloo et al., 2013) and increase the daily weight gain of young chickens without affecting feed intake (Graber et al., 1970). Proline also increased the villi height, small intestine weight and growth performance of weaned piglets (Wu et al., 1996). Proline can be converted into hydroxyproline through a series of biochemical reactions in organisms. The growth performance and feed utilization of spotted drum can be improved by adding hydroxyproline to the diet (Rong et al., 2020). The addition of proline or hydroxyproline to a plant protein-based diet is beneficial to the growth and muscle quality of yellow croaker (Wei et al., 2016, 2020) and proper supplementation of hydroxyproline in diet promoted muscle production and collagen synthesis, and promoted the growth of carp (Song et al., 2022). These results are consistent with the findings of this study that adding an appropriate amount of proline to the feed could enhance the growth performance of white shrimp, and are similar to the traditional view that proline is an amino acid indispensable for the body's growth and development (Baker, 2009; Ball et al., 1986; Graber et al., 1970; Wu et al., 2011). But it was found that adding proline to feed containing 15% fish meal had no significant effect on the growth performance of white shrimp (Xie et al., 2015). And the addition of proline to the feed containing 18.5% fish meal had no significant effect on the growth of salmon (Aksnes et al., 2008). It was reported that concentration of proline in rainbow trout muscle tissue is affected by the level of proline in the feed, and the endogenous synthesis rate of proline is insufficient (Zhang et al., 2006). This indicates that the impact of dietary proline on growth is influenced by various factors including fish species, feed composition, fish size, and supplementation concentration. Based on quadratic regression analysis of FCR and PER, 2.50% to 2.51% proline is recommended for white shrimp.

Collagen ranks first in terms of abundance in the human body's proteins, while glycine, proline and hydroxyproline collectively constitute 57% of collagen's amino acid composition (Li and Wu, 2018). Moreover, proline and glycine can be used as amino acids for cryoprotectants to protect cells or tissues from damage that occurs during freezing and storage, and hydroxyproline and glycine are pivotal in immune response and nutrient utilization (Aksnes et al., 2008; Wu et al., 2009). Proline in most mammals can be synthesized from glutamic acid, ornithine, arginine, and proline can be used as substrates to synthesize hydroxyproline and glycine (Wu et al., 2009). Simply put, hydroxyproline can be synthesized by proline in collagen by hydroxylation of prolyl-4-hydroxylase or prolyl-3-hydroxylase, and glycine can be synthesized by hydroxyproline under the action of enzymes such as 4-hydroxyproline oxidase (Wu et al., 2011; Xie et al., 2014). However, recent findings have shown that endogenous synthesis of proline, hydroxyproline and glycine is not sufficient to achieve maximum growth rates, optimize collagen production, or improve feed efficiency in fish, pigs, and chickens. Therefore, the optimal dietary concentration of proline must be determined to meet these nutritional requirements (Hou and Wu, 2017; Li and Wu, 2018). In this study, dietary proline supplementation elevates muscle levels of proline, hydroxyproline, and glycine in shrimp, paralleling findings in piglets (Wang et al., 2021), white shrimp (Xie et al., 2015), and carp (Song et al., 2022). This suggests dietary proline may enhance hydroxyproline (via hydroxylation) and glycine (via enzymatic conversion) synthesis, though further investigation is required to clarify metabolic pathways and nutritional requirements.

TOR, a critical regulator of cellular growth and metabolism, is central to health, disease, and aging through its sensing and integration of environmental signals (Kim et al., 2013; Laplante and Sabatini, 2012). Research shows proline activates the TOR signaling pathway to enhance protein synthesis by phosphorylating its downstream target S6. This mechanism promotes collagen production in rat hepatocytes and lung myofibroblasts (Ge et al., 2018; Oka et al., 2013), stimulates muscle protein synthesis in piglets (Wu et al., 2009), and facilitates epithelial differentiation in embryonic stem cells (Washington et al., 2010). Additionally, proline improves WM35 cell survival and reduces oxidative damage by activating the Akt/mTOR pathway (Natarajan et al., 2012). In this study, we analyzed TOR-related genes and proteins. The present study demonstrated that with the increase of dietary proline, the mRNA levels of *s6k*, *akt*, and *tor* increased significantly, and dietary proline supplement could significantly upregulate the expression of Akt protein. This is consistent with previous research that amino acids can increase protein production in cells by activating the Akt-TOR signaling pathway (Han et al., 2008; Ijichi et al., 2003; Tato et al., 2011; Zhao et al., 2020). The findings of this study demonstrate for the first time that moderate amounts of dietary proline can markedly enhance growth performance, feed utilization, and protein efficiency of white shrimp, possibly by increasing the activation of TOR pathway to improve these parameters.

Temperature is a crucial natural factor that influences the defense mechanisms of crustaceans, and hemocytes are key players of the immune system in shrimps (Koiwai et al., 2021; Qiu et al., 2011). The results of this research indicated that the proportion of granular cells did not change significantly after acute cold stress, while the proportion of hyaline cells decreased significantly, and the proportion of semi-granular cells, ROS, and apoptosis levels increased significantly. Similar results have also been reported in shrimp, where after cold shock in water at 13 °C, the proportion of semi-granular cells in white shrimp increased (Xu et al., 2019). The content of hyaline cells in hemocyte of tiger shrimp significantly decreased with the decrease of temperature (Wang and Chen, 2006). In addition, cold can lead to the generation of ROS and increase cell apoptosis in living organisms, while excessive ROS can threaten the survival of animals (Bozzo et al., 2017). According to reports, with the increase of salinity, the total free proline content of Chinese mitten crabs (*Eriocheir sinensis*), stone crabs (*Menippe mercenaria*), and blue crabs (*Callinectes sapidus*) significantly increased (Burton, 1992; Gilles and Schoffeniels, 1969; Robert, 1978). Proline has antioxidant properties because it can scavenge free radicals to cope with cellular oxidative stress (Wu et al., 2011), which may suggest that proline plays a vital role in slowing down the stress response of crustaceans. The results of this study showed that under acute low-temperature stress, adding an appropriate amount of proline increased the proportion of semi-granular cells, while reducing the proportion of hyaline cells, ROS, and cell apoptosis levels, which indicates that proline may help improve the ability of white shrimp to resist low-temperature stress. This is consistent with the research that proline could reduce intracellular ROS levels and cell apoptosis in mammalian cells, and that pyrroline-5-carboxylate dehydrogenase reduced ROS and apoptosis in drosophila S2 cells under Cd^2+^ stress (Krishnan et al., 2008; Liang et al., 2019; Liu et al., 2021; Schworer et al., 2020).

Additionally, to maintain metabolic balance in the body, shrimp can also eliminate ROS by activating their defense system to cope with oxidative stress. The defense system of shrimp includes some antioxidant enzymes (GPX, CAT, and SOD) and other non-enzymatic antioxidants, which can effectively alleviate oxidative stress (Tu et al., 2012; Xu et al., 2018). When shrimp face stress, they can also slow down damage by inducing the expression of oxidative protection genes, such as *gpx* and *cat* (Chang et al., 2009; Qian et al., 2012). Additionally, HSP70, as a molecular chaperone, blocked oxidative damage caused by external environmental stress through multiple mechanisms in white shrimp, and subsequently prevented apoptosis (Guan et al., 2021). Previous studies have shown that proline reacts with H_2_O_2_, singlet oxygen (^1^O_2_), and OH• (pH 7-8), leading to the formation of stable free radical adducts of proline, which subsequently results in a decrease in ROS level, an augmentation in total GSH, and regulation of the intracellular redox balance (Kaul et al., 2008; Liang et al., 2013; Liu et al., 2021). The results of this study indicate that cold significantly increased the expression of antioxidant related genes (*gpx*, *sod*, *cat*, and *hsp70*), and dietary proline supplement significantly increased the mRNA expression of *gpx* and *hsp70*, enhanced antioxidant capacity, reduced ROS and alleviated oxidative stress. This is consistent with research showing that dietary proline improved the stress resistance of abalone (Vosloo et al., 2013) and the antioxidant capacity of yellow croaker (Wei et al., 2020).

Apoptosis is programmed cell death, through which the body can clear damaged cells, thereby avoiding the leakage of intracellular hydrolytic enzymes and cell damage caused by tissue rupture (Malhi et al., 2006). Apoptosis involves the activation of a large number of catabolic enzymes, such as proteases caspase-3 and caspase-9, which can form a protective membrane during the process of cell apoptosis to prevent the efflux of intracellular hydrolytic enzymes, having a critical impact on different stages of cell apoptosis (Chang et al., 2008; Green, 2005). Romol is a reactive oxygen species regulatory factor and its gene expression was induced by microcystin injection, leading to the accumulation of ROS and hemocyte apoptosis (He et al., 2017). P53 protein, as a phosphor nuclear transcription factor, can further regulate the expression of genes involved in the integrated cellular pathways, such as apoptosis, in response to environmental perturbations (Levine, 2020). In this study, acute low-temperature stress significantly increased the mRNA expression of apoptosis related genes (*caspase3*, *caspase9*, *romo1* and *p53*), while appropriate dietary proline significantly reduced the expression of apoptosis related genes in the hepatopancreas. Proline accumulation is an important metabolic adaptive mechanism in many organisms under biotic and abiotic stresses (Hu et al., 2008; Rizzi et al., 2015). Proline can effectively protect mammalian cells from oxidative stress by regulating intracellular redox environment, inhibiting reactive oxygen species levels and reducing apoptosis (Krishnan et al., 2008). When proline was added and WM35 cells were subjected to hydrogen peroxide stress, the protective effect of proline involved activation of the Akt signaling pathway and phosphorylation of FoxO3a, which in turn enhanced cell viability possibly by inhibiting FoxO3a-induced cell death (Natarajan et al., 2012). In *L.* *vannamei*, HSP70 was found to block the ROS-induced apoptotic pathway triggered by external environmental stress through multiple mechanisms (Guan et al., 2021). The present study showed that proline supplement reduced apoptosis by decreasing ROS production, as well as caspase 3 and caspase 9 expression.

Autophagy, a tightly regulated intracellular recycling process in eukaryotes, evolved through environmental-biological adaptation interplay (Tian et al., 2015). Normally inactive under homeostasis, it responds to stressors like nutrient deprivation, hypoxia, and infections by degrading cellular components to enhance survival (Kroemer et al., 2010). In mammals, proline metabolic enzymes can activate redox sensitive pathways to regulate apoptosis and autophagy, promoting cell survival (Liu et al., 2005, 2006; Maxwell and Davis, 2000; Phang et al., 2015; Zabirnyk et al., 2010). The present study revealed a significant increase in the mRNA expression of autophagy related genes (*lc3*, *atg4*, *becline-1*, and *atg1*) in response to cold stress, and these genes significantly increased with increasing dietary proline levels. It has been reported that aquatic animals enhanced autophagy under low-temperature stress, which may be related to AMPK/TOR signaling pathway (Liang et al., 2021; Lu et al., 2018; Wu et al., 2019), and proline catabolic enzymes can act as downstream activators of AMPK, promoting autophagy or promoting tumor cell survival through ATP production (Liu and Phang, 2012). It has been reported that proline in hepatopancreas of white shrimp gradually increased as the ambient temperature declined from 28 to 13 °C (Zhou et al., 2011), and autophagy promoted proline biosynthesis in cancer-associated fibroblasts (Bai et al., 2023). Although autophagy has been a hot topic in scientific research recently, research on the mechanism of how autophagy maintains the survival of crustaceans under low-temperature stress is still relatively lagging behind, and the mechanism of proline regulating autophagy still needs further research.

TOR and AMPK are two key kinases for organisms to sense nutrient and energy levels, regulate metabolic balance and cell growth, and they are also key upstream regulators of cell autophagy (Li et al., 2021; Mariño et al., 2014). When cells are under stress, AMPK activation can directly phosphorylate the downstream key protein raptor, thereby transmitting nutritional signals to TORC1 to inhibit cell growth and the synthesis of biomolecules (Gwinn et al., 2008). For example, inhibiting the effector protein S6K downstream of the TORC1 pathway can save energy (Ishizuka et al., 2013; Schalm et al., 2003). It has been reported that the transcriptional level of *tor* in white shrimp significantly increased at 1.5 and 3 h after 12 °C low-temperature stress, but decreased at 12 and 24 h (Liang et al., 2020). In this study, the expression of the *tor* gene did not show significant changes. It is speculated that this might be because the stress temperature and time point we chose were possibly in the transitional stage of the dynamic change of the gene expression quantity (between the initial rising period and the subsequent falling period), thus failing to capture the significant differences. In this study, cold stress significantly increased the mRNA expression levels of *ampk* and *raptor*, while significantly reduced the mRNA expression levels of downstream key genes *s6k* and *4e-bp* in the TOR signaling pathway. Research has shown that apoptosis and autophagy can be elicited by a common upstream signal, and the body can adapt to changing environments by regulating the balance between cell autophagy and apoptosis (Maiuri et al., 2007). This suggests that white shrimp may regulate autophagy and apoptosis through the AMPK/TOR signaling pathway, thereby reducing synthetic metabolism to maintain energy supply under environmental pressure conditions and mitigating damage caused by cold stress. Research has shown that proline regulated TORC1 signaling in porcine trophectoderm cells and regulated the intracellular redox environment through the enhancement of proline transport (Liu et al., 2021). Under cold stress, the findings of this research suggest that dietary proline significantly reduced the mRNA level of *s6k*, while significantly increased the mRNA expression of raptor and *ampk*, and the protein level of p-raptor. Research findings indicate that the regulation of intracellular free amino acid concentration is crucial for the adaptation to salinity changes in crustaceans and various other marine invertebrate taxa (Burton, 1992; Gilles and Schoffeniels, 1969). Only a few non-essential amino acids, such as proline, alanine, and glycine, are the main contributors to the response, and exhibit rapid and quantitatively significant concentration changes with changes in environmental salinity. Proline accumulation serves dual roles as both a stress adaptation and antioxidant defense (Krishnan et al., 2008), while its mitochondrial metabolism through Pro dehydrogenase (ProDH) produces approximately 30 ATP molecules per oxidized L-proline molecule (Zarse et al., 2012), providing critical energy during nutrient scarcity (Hare and Cress, 1997; Phang et al., 2008, 2010). Burton has shown that under conditions of high osmotic stress, there is a notable augmentation in the detectable synthesis of proline within the euryhaline intertidal copepod *Tigriopus californicus*. But after about 24 h of accumulation of proline, the synthesis of proline was effectively shut down, so supplementing proline was crucial (Burton, 1986, 1991a, 1991b). And research has shown that producing protein and endogenous proline are expensive in terms of energy, and endogenous proline is produced from glutamate by consuming nicotinamide adenine dinucleotide/nicotinamide adenine dinucleotide phosphate (NADH/NADPH) enzymes or ATP , and the mechanism of proline accumulation under stress conditions may include protein degradation, uptake from culture medium, and de novo synthesis (Burton, 1992; Loayza-Puch and Agami, 2016). Recent studies have shown that exogenous proline can activate pTr2 p-AMPKα (Liu et al., 2021), and the activation of AMPK can decrease the protein levels of p-mTOR, p-S6, and p-4EBP1 (Chen et al., 2010; Li et al., 2010). Therefore, this results indicate that proline may activate the AMPK signaling pathway to phosphorylate downstream key protein raptor and inhibit TORC1, reduce synthetic metabolism, maintain energy supply under environmental pressure conditions, and so as to alleviate the damage caused by cold stress on white shrimp.

## 5 Conclusion

In conclusion, the present study showed that 2.50% to 2.72% dietary proline significantly improved growth performance, feed utilization and protein synthesis by activating TOR signal pathway under 30 °C feeding trial. In addition, under cold stress, 2.60% to 2.79% dietary proline significantly decreased ROS levels, cell apoptosis and the expression of apoptosis related genes, but increased the expression of genes involved in antioxidant and autophagy. White shrimp may regulate cell apoptosis and autophagy by inhibiting the TOR signal pathway, thus reducing the damage caused by cold stress. For the first time, this study reveals that dietary proline improves protein synthesis and cold resistance in white shrimp by the TOR signaling pathway, which helps us to understand the mechanism of cold stress and nutritional regulation in crustaceans.

## Credit Author Statement

**Danyang Zou:** Writing – original draft, Methodology, Investigation, Formal analysis, Data curation. **Ming Yang:** Writing – original draft, Methodology, Formal analysis, Data curation. **Zhiwen Chen:** Validation, Investigation. **Pei Lin:** Visualization, Data curation. **Yingtao Li:** Investigation, Data curation. **Xingyuan Liu:** Visualization, Formal analysis. **Beiping Tan:** Resources. **Chaoxia Ye:** Writing – review & editing, Supervision, Resources, Conceptualization.

## Declaration of competing interest

We declare that we have no financial and personal relationships with other people or organizations that can inappropriately influence our work, and there is no professional or other personal interest of any nature or kind in any product, service and/or company that could be construed as influencing the content of this paper.
