# Supplementary material for: Effects of proline on growth performance, protein synthesis and cold resistance in white shrimp (Litopenaeus vannamei)
Source: Anim Nutr. 2025 Jul 30;23:220–34. doi: 10.1016/j.aninu.2025.06.002 (PMC12664086; doi:10.1016/j.aninu.2025.06.002)
Supplement: Multimedia component 1 [file mmc1.docx]

**Table S1**

The primer sequences used for real time PCR.

| Genes | Full names | Forward primer (5-3') | Genbank accession no. | Function | Length, bp |
| --- | --- | --- | --- | --- | --- |
| *Ef1α* | Elongation factor 1-alpha-like | F-TGGCTGTGAACAAGATGGAC | XM_027373349.1 | Internal control | 20 |
|  |  | R-AGATGGGGATGATTGGGACC |  |  | 20 |
| *Tor* | Target of rapamycin | F-TGCCAACGGGTGGTAGA | [XM_027372359.1](https://www.ncbi.nlm.nih.gov/entrez/viewer.fcgi?db=nucleotide&id=1536077780) | TOR pathway | 17 |
|  |  | R-GGGTGTTTGTGGACGGA |  |  | 17 |
| *Raptor* | Regulatory associated protein of TOR | F-CTGCTTTCCAGGCTACTC | [XM_027360909.1](https://www.ncbi.nlm.nih.gov/entrez/viewer.fcgi?db=nucleotide&id=1536056255) | TOR pathway | 17 |
|  |  | R-TCACAATCCAAGGTCCAG |  |  | 18 |
| *S6k* | Ribosomal protein S6 kinase | F-GCAAGAGGAAGACGCCATA | [XM_027368997.1](https://www.ncbi.nlm.nih.gov/entrez/viewer.fcgi?db=nucleotide&id=1536071408) | TOR pathway | 19 |
|  |  | R-CCGCCCTTGCCCAAAACCT |  |  | 19 |
| *4e-bp* | Eukaryotic initiation factor 4E-binding proteins | F-ATGTCTGCTTCGCCCGTCGCTCGCC | [XM_027367939.1](https://www.ncbi.nlm.nih.gov/entrez/viewer.fcgi?db=nucleotide&id=1536069428) | TOR pathway | 25 |
|  |  | R-GGTTCTTGGGTGGGCTCTT |  |  | 19 |
| *Akt* | Protein kinase B | F-AAATGACTATGGACGAGGTGTT | [XM_027364781.1](https://www.ncbi.nlm.nih.gov/entrez/viewer.fcgi?db=nucleotide&id=1536063467) | TOR pathway | 22 |
|  |  | R-GTTGATGGTGATGTAGAAGGGG |  |  | 22 |
| *Ampk* | AMP-activated protein kinase | F-GCGCTTTGCTGATGCTAATGCC | [XM_027375044.1](https://www.ncbi.nlm.nih.gov/entrez/viewer.fcgi?db=nucleotide&id=1536082751) | TOR pathway | 20 |
|  |  | R-GGGTGTGGCCTGAATGGAACTG |  |  | 21 |
| *Cat* | Catalase | F-TACTGCAAGTTCCA TTACAAGACG | [XM_027383088.1](https://www.ncbi.nlm.nih.gov/entrez/viewer.fcgi?db=nucleotide&id=1536041747) | Antioxidation | 24 |
|  |  | R-GTAA TTCTTTGGATTGCGGTCA |  |  | 22 |
| *Sod* | Superoxide dismutase | F-GCAATGAATGCCCTTCTACC | [XM_027376216.1](https://www.ncbi.nlm.nih.gov/entrez/viewer.fcgi?db=nucleotide&id=1536084915) | Antioxidation | 20 |
|  |  | R-CAGAGCCTTTCACTCCAACG |  |  | 21 |
| *Gpx* | Glutathione peroxidase | F-GGCACCAGGAGAACACTAC | [XM_027372127.1](https://www.ncbi.nlm.nih.gov/entrez/viewer.fcgi?db=nucleotide&id=1536077323) | Antioxidation | 20 |
|  |  | R-CGACTTTGCCGAACATAAC |  |  | 20 |
| *Hsp70* | Heat shock protein 70 | F-CCTCCAGGACTTCTTCAACG | [XM_027369405.1](https://www.ncbi.nlm.nih.gov/entrez/viewer.fcgi?db=nucleotide&id=1536072169) | Apoptosis | 21 |
|  |  | R-GGTCACGTCCAACAGCAAC |  |  | 21 |
| *Caspase-3* | Cysteinyl aspartate specific proteinase-3 | F-ACGAGAAGTCGCCAGGAGGT | [XM_027352650.1](https://www.ncbi.nlm.nih.gov/entrez/viewer.fcgi?db=nucleotide&id=1536035967) | Apoptosis | 22 |
|  |  | R-CGGTCGCATTGTGATGATAAAA |  |  | 22 |
| *Caspase-9* | Cysteinyl aspartate specific proteinase-9 | F-AGGGCAAGCCGAAGATTT | [XM_027358108.1](https://www.ncbi.nlm.nih.gov/entrez/viewer.fcgi?db=nucleotide&id=1536051035) | Apoptosis | 19 |
|  |  | R-CGGTGGTGCTGTAGATAACC |  |  | 20 |
| *Romo1* | Reactive mitochondrial oxygen species modulator 1 | F-ATTTATGATGGGTTTCTCAGTCGG | [XM_027366490.1](https://www.ncbi.nlm.nih.gov/entrez/viewer.fcgi?db=nucleotide&id=1536066698) | Apoptosis | 23 |
|  |  | R-TTCCCTACATTATTCATTAACTCCCT |  |  | 26 |
| *P53* | P53-related protein kinase | F-CGAATCCCCACATCCACG | [XM_027365892.1](https://www.ncbi.nlm.nih.gov/entrez/viewer.fcgi?db=nucleotide&id=1536065545) | Apoptosis | 18 |
|  |  | R-TGCCGAATTTGTGACGACCT |  |  | 20 |
| *Lc3* | Microtubule-associated proteins 1a and 1b | F-CCTCTCCTCGACAAGACCAA | [XM_027378530.1](https://www.ncbi.nlm.nih.gov/entrez/viewer.fcgi?db=nucleotide&id=1536089221) | Autophagy | 20 |
|  |  | R-TCATGGTGGTGGTGTTCGTA |  |  | 19 |
| *Atg4* | Autophagy related 4 homolog d | F-CCTCTGCCAAGATGCTGTGA | [XM_027358027.1](https://www.ncbi.nlm.nih.gov/entrez/viewer.fcgi?db=nucleotide&id=1536050885) | Autophagy | 19 |
|  |  | R-GAAGGGTCCATGCGAGAGAG |  |  | 20 |
| *Atg1* | Autophagy related protein 1 antibody | F-CGCTCGCTACTGACACGCTAC | [XM_027353707.1](https://www.ncbi.nlm.nih.gov/entrez/viewer.fcgi?db=nucleotide&id=1536042781) | Autophagy | 20 |
|  |  | R-TCTCCTGTGCCTACGCTTCATTTG |  |  | 24 |
| *Beclin-1* | Coiled-coil, myosin-like B cell lymphoma 2 family protein interacting protein | F-TGTGGAAGGTGGTGTTGAA | [XM_027361370.1](https://www.ncbi.nlm.nih.gov/entrez/viewer.fcgi?db=nucleotide&id=1536057107) | Autophagy | 20 |
|  |  | R- GAGGAGAAAGCACGATTGG |  |  | 19 |

F = forward; R = reverse.
